# Chinas Belt and Road Initiative wandelt sich

**DOI:** 10.1007/s10273-021-3049-4

**Published:** 2021-11-18

**Authors:** Britta Kuhn

**Affiliations:** Wiesbaden Business School der Hochschule RheinMain, Bleichstr. 44, 65183 Wiesbaden, Deutschland

**Keywords:** F21, O22, O53

## Abstract

Schon vor der Corona-Pandemie belasteten finanzielle und geopolitische Probleme die „Neue Seidenstraße“: Chinas Gesamtinvestitionen gehen seit Jahren zurück, wichtige Bauprojekte sind unrentabel, die Partner in Ost- und Südosteuropa wenden sich ab, und umweltschädliche Vorhaben stoßen zunehmend auf Widerstand. Seit 2020 bieten wohlhabende Demokratien verstärkt Alternativen, und auch die Volksrepublik stundet Schulden. Xi Jinping forciert daher neben dem „BRI Green Partnership“ vor allem die „Digital Silk Road“ und „China Standards 2035“, also globale Technologie- und Normierungsprojekte.

## Abstract (en)

Even before the coronavirus pandemic, financial and geopolitical problems were weighing on the “New Silk Road”: China’s overall investment has been declining for years, key construction projects are unprofitable, partners in Eastern and Southeastern Europe are turning away, and environmentally harmful projects are increasingly meeting resistance. Since 2020, affluent democracies reinforce alternatives and the People’s Republic also defers debt. In addition to the “BRI Green Partnership”, Xi Jinping is therefore pushing the “Digital Silk Road” and “China Standards 2035”, i. e. global technology and standardisation projects.

Wie groß ist Xi Jinpings weltumspannendes Infrastrukturprojekt, die Belt and Road Initiative (BRI)? Die Antworten variieren erheblich mit den genutzten Quellen, einbezogenen Ländern und Investitionsvorhaben. So schätzt die Weltbank auf Basis einer chinesischen Datenbank, dass bis 2019 rund 575 Mrd. US-$ in 70 Länder flossen – die Volksrepublik nicht mitgerechnet. Davon hätten die vier Transitländer Russland, Pakistan, Malaysia und Indonesien die Hälfte erhalten. Absoluter Spitzenreiter sei die Russische Föderation mit über 100 Mrd. US-$ gewesen (Ruta, 2019). Der Finanzanalyst Refinitiv (2020) kommt mit einer eigenen Datenbank bis Mitte 2020 auf insgesamt 2,3 Bio. US-$ und benennt ebenfalls Russland als Hauptzielland der BRI – mit insgesamt 258 Mrd. US-$. Das American Enterprise Institute, dessen Datenbasis am transparentesten ist, errechnet bis Mitte 2021 Gesamtinvestitionen von 795 Mrd. US-$ in 115 Ländern (AEI, 2021). Nach dieser Aufstellung repräsentieren die vier genannten Empfängerstaaten ein Fünftel aller Investitionen. Russland steht hier nur für 37 Mrd. US-$ und stattdessen Pakistan an Platz eins der Empfänger.

So unterschiedlich diese Schätzungen, so einhellig ist die Erkenntnis, dass die jährlichen Investitionen schon vor der Corona-Pandemie sanken (vgl. Abbildung 1). Speziell die beiden staatlichen Entwicklungs- bzw. Außenhandelsbanken CDB und ExIm stellten von 2013 bis 2019 insgesamt 281 Mrd. US-$ bereit. 2016 gipfelten ihre Zuwendungen bei 75 Mrd. US-$. Danach gingen sie kontinuierlich zurück und lagen 2019 bei insgesamt 4 Mrd. US-$ (BU Global Development Policy Center). Die Direktinvestitionen der Volksrepublik entwickelten sich analog: Sie erreichten 2016 ihren Höhepunkt und kamen speziell in der EU 2019 nur noch auf ein Drittel ihres Niveaus von 2016 (Kratz et al., 2020a).

## Unrentable Bauprojekte

Schon 2019 wirkten wichtige Einzelmaßnahmen der BRI finanziell und geopolitisch bedenklich (Kuhn, 2019). Seither kam detailliertes Anschauungsmaterial hinzu und die Neue Seidenstraße wird zunehmend kritisch begleitet (z. B. Hillman, 2020; Heilmann, 2020). Stellvertretend sei die jüngere Entwicklung in Pakistan zusammengefasst: Die Atommacht ist eine überragend wichtige Seidenstraßen-Partnerin Chinas, spielt eine zentrale Rolle im Afghanistan-Konflikt und befindet sich mit Indien im Dauerzwist. Die chinesisch-pakistanischen Energieversorgungs- und Transportprojekte erreichten ihren Höhepunkt bereits 2015 mit 22,4 Mrd. US-$. Zum Vergleich: Chinas Gesamtinvestment in Afghanistan lag bis Mitte 2021 bei nur 210 Mio. US-$ – für ein Transportvorhaben 2017 (AEI, 2021).[1] Allein die Kosten für eine Landverbindung von China nach Gwadar am Arabischen Meer explodierten von ursprünglich geplanten zehn bis 20 auf inzwischen mindestens 62 Mrd. US-$. Dieser „China-Pakistan Economic Corridor" (CPEC) dürfte nun frühestens 2030 fertig werden. Ausgerechnet an der Grenze zum Iran, nämlich in der umkämpften Krisenprovinz Belutschistan, soll der Meerestourismus mit chinesischen Geldern erblühen. Vollendete Projekte verspäteten und verteuerten sich oft gegenüber der Planung (Mardell, 2020a). Das lag – neben Korruption, Terrorangriffen und Bürokratie – auch an erhebli-

1 Ob der Rückzug des Westens Beijings Engagement in Afghanistan begünstigen oder bremsen dürfte, erscheint nicht ausgemacht.

**Prof. Dr. Britta Kuhn** lehrt Volkswirtschaftslehre an der Wiesbaden Business School der Hochschule RheinMain.

Abbildung 1

**BRI-Gesamtinvestitionen pro Jahr und Region**

in Mrd. US-$

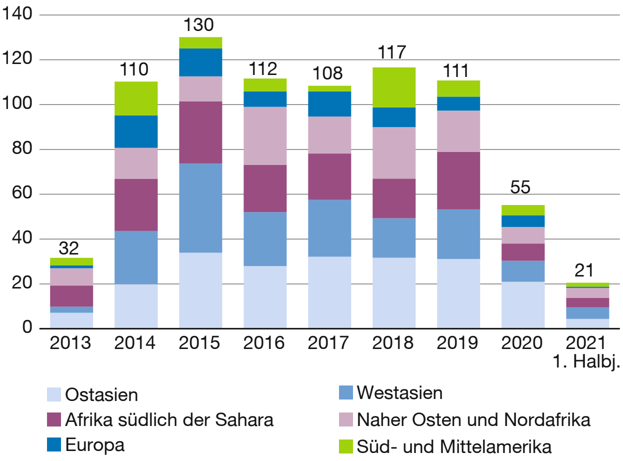

Anmerkung: Ohne Investitionen in China.

Quelle: eigene Darstellung nach AEI (2021)

chen wirtschaftlichen Problemen: Die Corona-Pandemie bescherte Pakistan 2020 die zweite Zahlungsbilanzkrise seit 2018. Premierminister Khan erwirkte 2019 ein erstes Rettungspaket des Internationalen Währungsfonds (IWF) von umgerechnet 6 Mrd. US-$ für gut drei Jahre. Im April 2020 kam eine weitere IWF-Hilfe von rund 1,4 Mrd. US-$ hinzu. Khan stutzte nicht nur CPEC, sondern auch die sino-pakistanischen Pläne für eine industrielle Zusammenarbeit und für Sonderwirtschaftszonen. Statt weiterer Infrastrukturanlagen strebt der Premier Schuldenschnitte an und deckt Fehlkalkulationen auf. 2020 bestätigte eine von ihm beauftragte Kommission z. B., dass die chinesischen Vertragspartner für zwei Kohlekraftwerke 3 Mrd. US-$ zu viel berechnet hätten. Teure Prestigeprojekte wie der Hafen in Gwadar oder die Zugverbindung zwischen Peschawar und Karatschi laufen jedoch weiter (Lew et al., 2021; The Economist, 2020; IMF, 2019; IMF, 2021a).

Selbst in reichen Ländern mit widerstandsfähigen Institutionen erweisen sich die meisten öffentlichen Großprojekte als zu teuer, verspätet und weniger nützlich als erwartet: Bestenfalls acht von 1.000 liefern ihren versprochenen Nutzen innerhalb des Zeit- und Kostenrahmens. Die Mehrkosten von 31 bekannten Investitionsvorhaben in wohlhabenden OECD-Staaten betrugen sogar bis zu 1.600 % der ursprünglich veranschlagten Summe (Flyvbjerg, 2017). Chinas Infrastrukturanlagen konzentrieren sich indes auf fragile Entwicklungs- und Schwellenländer. Selbst eine Studie der Weltbank benennt in diesen Ländern fiskalische, politisch-institutionelle, umwelt- und sozialpolitische sowie Governance-Probleme inklusive Korruption bei der Umsetzung der Seidenstraßen-Initiative (Ruta, 2019). Das ist bemerkenswert, weil diese Untersuchung ganz überwiegend die Vorteile der BRI betont, namentlich niedrigere Transport- und folglich Handelskosten. Eine andere Weltbank-Untersuchung relativiert sogar diese Transportgewinne: Nur 29 von 68 Transportprojekten verbesserten in 23 eurasischen Seidenstraßen-Ländern inklusive China die Verbindung. Der geschätzte Anteil erfolgreicher Vorhaben steigt allerdings mit einem Kunstgriff von 29 auf 43: Nämlich unter der Annahme, dass alle komplementären Projekte bereits fertiggestellt wären (Reed und Trubetskoy, 2019).

## Nachlassende 17+1-Initiative

Chinas Kooperation mit Süd- und Osteuropa bereitet Teilen der EU seit Jahren Sorgen. Schrittweise übernahm z. B. die chinesische Reederei COSCO den Athener Hafen (z. B. Naß, 2021). In die 17 Partnerstaaten, darunter zwölf EU-Mitglieder und fünf Länder des Westbalkans, flossen bis Mitte 2021 insgesamt 26,9 Mrd. US-$. Das entsprach 3,4 % aller Investitionen, die der Neuen Seidenstraße zugerechnet werden. Hauptempfänger war Serbien, wo China noch im April 2021 investierte. Überdies starteten in Ungarn, Polen und Bosnien-Herzegowina trotz Corona neue Projekte. In den weiteren Zielländern hielten sich die konkreten Aktivitäten zeitlich oder mengenmäßig zuletzt in Grenzen. In Albanien, Estland, Litauen und der Slowakei flossen bisher keine finanziellen Mittel (vgl. Abbildung 2).[2] Anfang 2021 setzten Slowenien, Kroatien, Rumänien und die Tschechische Republik öffentliche Ausschreibungen aus, an denen chinesische Unternehmen beteiligt waren. Dies betraf vor allem Kernkraftwerke, Autobahnen und Bahnlinien. Neben schlechter Qualität spielten nationale Sicherheitsbedenken und politischer Druck aus den USA eine wachsende Rolle. Rumänien will chinesische Firmen künftig sogar komplett von öffentlichen Ausschreibungen ausschließen. Dies plant auch Litauen (Brüggmann, 2021).[3]

## Widerstand gegen umweltschädliche Investitionen

Die Umweltfolgen von Energie- und Transportprojekten entlang der Seidenstraße sind zwiespältig: Transportprojekte senken die Umweltverschmutzung beispielsweise nur, soweit Schienen den Straßenverkehr ersetzen. Oft führen diese Investitionen aber zu einem steigenden Verkehrsaufkommen, höheren $CO_2$-Emissionen sowie zu

2 Nach Ajeti (2020) investierte China in Albanien umfangreich, beispielsweise im Autobahnbau. In der Datenbank des AEI (2021) zu den konkreten Seidenstraßen-Projekten taucht Albanien dagegen nicht auf.

3 Italien gehört der 17+1-Unternehmung nicht an, sondern unterzeichnete 2019 als erstes großes Industrieland eine Rahmenvereinbarung zur Beteiligung an der Neuen Seidenstraße (Naß, 2021). Unter Ministerpräsident Draghi kühlte die Beziehung zu China ab. Gleiches gilt für Mitsotakis‘ Griechenland.

Abbildung 2

**BRI-Investitionen in den 17+1-Ländern bis Mitte 2021**

in Mio. US-$

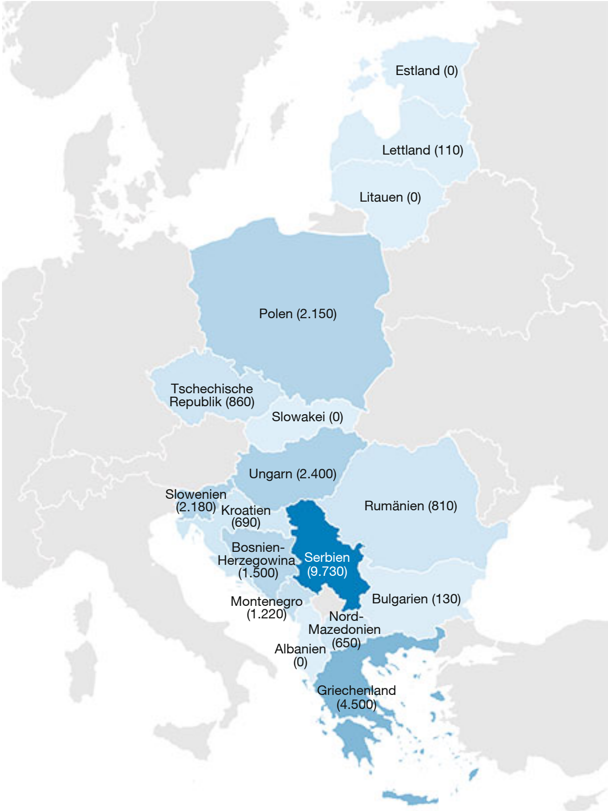

Quelle: eigene Darstellung nach AEI (2021).

topografischen und hydrologischen Beeinträchtigungen (Ruta, 2019). Für ausländische Energieanlagen gab die Volksrepublik laut einer unveröffentlichten chinesischen Analyse 2020 insgesamt 20 Mrd. US-$ aus. Davon flossen 57 % in erneuerbare Energien, aber zugleich 27 % in Kohleanlagen. Fraglich ist dabei z. B., ob alle Wasserkraft-Vorhaben nachhaltig sind. Zwar stoßen sie selbst kein Kohlendioxid aus, können aber ganze Ökosysteme zerstören. Unter anderem Ägypten, Pakistan, Bangladesch und Vietnam sehen deshalb ihre bisherige Zusammenarbeit mit China kritisch (Shepherd, 2021). Auch ist bei der Interpretation chinesischer Datenbanken Vorsicht geboten: Refinitiv (2020) listet z. B. unter den sechs größten BRI-Projekten im Bereich erneuerbarer Energien fünf Atomkraftwerke. Darunter befinden sich zwei britische Anlagen, obwohl Großbritannien keine Seidenstraßen-Kredite erhält, sondern chinesische Direktinvestitionen.

## Reaktionen wohlhabender Demokratien

Chinas Kredite an fragile Staaten erhalten zunehmend Konkurrenz: Australien startete 2019 eine Finanzierungsfazilität für Infrastrukturprojekte im pazifischen Raum. Auch die USA schufen 2019 eine Organisation, die internationale Entwicklungsprojekte mit bis zu 1 Mrd. US-$ bezuschusst und private Finanzquellen einbezieht. Die USA, Japan und Australien gründeten 2019 das „Blue Dot Network“. Es fördert Infrastrukturprojekte in armen Ländern, um die dortigen Investitionslücken zu füllen. Sie werden auf 2,5 bis 3,5 Billionen US-$ geschätzt (AIFFP; DFC; OECD, 2021; Naß, 2021).

Seit 2020 unterstützt der IWF ärmere Mitgliedsregierungen mit zusätzlich rund 250 Mrd. US-$ zur umfassenden Bewältigung der Corona-Krise (IMF, 2021a). Im August 2021 gab der Fonds eine neue Kreditlinie historischen Ausmaßes frei. Von den insgesamt etwa 650 Mrd. US-$ werden allerdings „nur“ rund 275 Mrd. US-$ in Schwellen- und Entwicklungsländer fließen, da sie an die Länderquoten des IWF gekoppelt sind (IMF, 2021b). Der G7-Gipfel hatte wenige Wochen zuvor eine Absichtserklärung verabschiedet, die auf eine ehrgeizige globale Infrastrukturpartnerschaft zielt. Dafür soll die reiche Welt bis 2025 jährlich 100 Mrd. US-$ aus öffentlichen und privaten Quellen mobilisieren. Die sieben Regierungen fordern IWF, G20 und weitere Industrieländer auf, gemeinsam für sauberes Wachstum zu sorgen, die Partnerschaft mit Afrika zu vertiefen und die bedürftigsten Staaten stärker als bisher zu unterstützen (G7, 2021). Im Dunkeln bleibt unter anderem, welcher Anteil der Gelder in Schwellen- und Entwicklungsländer fließen soll. Gleichzeitig zum G7-Gipfel appellierten die Außenminister der EU an die Europäische Kommission, wirksame und sichtbare globale Projekte bis März 2022 umzusetzen (Rat der Europäischen Union, 2021). Deren bisherige „Konnektivitätsstrategie“ hatte nichts dergleichen hervorgebracht (Europäisches Parlament, 2020). Schließlich gerät Indien zunehmend in den Blick, nicht nur in der EU: Chinas großer Rivale, die USA, Japan und Australien werteten im März 2021 ihren vierseitigen Sicherheitsdialog „Quad“ auf. Dabei positionierten sie sich glasklar als Gegengewicht zu China zugunsten eines freien und offenen Indopazifiks. Den dortigen Anrainern stellten sie unter anderem einen besseren Zugang zu Impfdosen in Aussicht (The White House, 2021).

## Gestundete Schulden

Bis zur Corona-Krise war oft von einer Schuldenfalle die Rede, in der sich Chinas Kreditnehmende befänden. Abschreckendes Aushängeschild dieser Diskussion war der Tiefseehafen Hambantota: Sri Lanka konnte seine Schulden von mehr als 1 Mrd. US-$ nicht begleichen. China sicherte sich daraufhin die Nutzungsrechte für 99 Jahre – ein symbolträchtiger Zeitraum. Denn genauso lange hatte Großbritannien seinerzeit Hongkongs Neue Territorien vom damaligen chinesischen Kaiserreich gepachtet (Naß,

2021). Nach Kratz et al. (2020b) übernimmt China bei größeren Zahlungsproblemen indessen äußerst selten ausländische Vermögenswerte. Genauso wenig schreibe Peking die Schulden direkt ab. Sie würden in aller Regel neu verhandelt – sprich: gestundet. Daran habe sich seit Ausbruch der Corona-Krise nichts geändert. Dies entspricht dem gemeinsamen Vorgehen der G20 in der Pandemie. An deren Initiative zur Aussetzung des Schuldendienstes in 73 Entwicklungsländern beteiligt sich auch die Volksrepublik. Schätzungen, wie hoch Chinas gefährdete Außenstände insgesamt sind, variieren gleichwohl stark (Kaaresvirta und Laakkonen, 2021). Am oberen Ende kommen Horn et al. (2020) für 2019 mit offiziellen Zahlungsbilanzdaten auf chinesische Außenstände, die gut 1,5 % des weltweiten Bruttoinlandsprodukts ausmachen.[4] Sie überträfen damit die Guthaben der Weltbank oder sämtlicher Regierungen des Pariser Clubs zusammen. Die tatsächlichen Kredite schätzen die Autor:innen noch wesentlich höher ein.

### BRI-Ausrichtung auf Nachhaltigkeit, Digitalisierung und Normsetzung

Die umfassenden Nachhaltigkeitsziele der UN von 2015, die bis 2030 erreicht sein sollen, verabschiedete auch China (UNDP China, 2021). 2019 präzisierte Xi Jinping, dass Infrastrukturprojekte der Neuen Seidenstraße grün und nachhaltig sein sollten. Das Green Belt and Road Initiative Center (2021) plant und koordiniert entsprechende Maßnahmen. Vieles davon ist zukunftsorientiert, etwa ein Ampelsystem, bei dem umweltfreundliche Projekte günstigere Kreditkonditionen erhalten sollen (Refinitiv, 2020). Im Juni 2021 starteten 29 Mitgliedsländer der Seidenstraße das „BRI Green Partnership“. Es wird als Gegenentwurf zur G7-Initiative für weltweit sauberes Wachstum vermarktet und mit deren „B3W“-Plänen – was für „Build Back Better World“ steht – ausführlich verglichen (Nedopil Wang und Tang, 2021).

Die „Digital Silk Road“ (DSR) erklärte Xi Jinping ebenfalls 2019 zu einer Priorität. Laut Datenbank des Mercator-Instituts für Chinastudien (MERICS) hatte sein Land bis dahin entsprechende Projekte für insgesamt 17 Mrd. US-$ abgeschlossen. Sie umfassten die Bereiche Glasfaserkabel und Telekommunikationsnetze, E-Commerce sowie mobiles Bezahlen. Die Volumina erschienen zu diesem Zeitpunkt zwar klein im Vergleich zu Chinas weltweiten Energie- und Transportprojekten. Aber die Wachstumsdynamik beeindruckte. So plante die Volksrepublik z. B. schon vor der Corona-Krise zwischen Pakistan, Ostafrika und Europa weitverzweigte Glasfaserkabelverbindungen unter dem symbolträchtigen Kürzel „PEACE“. Eine entsprechende Verbindung zwischen Kamerun und Brasilien war bereits fertig (Eder et al., 2019). Der sprunghaft gestiegene Bedarf digitaler Infrastruktur seit der COVID-19-Pandemie hat die Digitale Seidenstraße in den Mittelpunkt der chinesischen Anstrengungen gerückt – zumal Digitalprojekte in der Regel kostengünstiger als traditionelle Bauvorhaben sind und Technologie eine Schlüsselrolle im geostrategischen Wettbewerb mit den USA spielt. Xi Jinping und Li Keqiang setzen sich daher seit 2020 z. B. intensiv für eine umfassende 5G-Netzabdeckung in China und den BRI-Partnerländern ein. Chinesische Unternehmen sind bekanntlich selbst in vielen liberalen Demokratien wichtige Partner beim 5G-Ausbau (Refinitiv, 2020; Arcesati, 2020). Noch mehr Erfolg dürfte Chinas Digitalstrategie in Autokratien wie Myanmar oder Russland ernten (Hao und Rawat 2019; Lazanyuk und Revinova, 2020).

Das Forschungsprojekt „China Standards 2035“ empfiehlt der chinesischen Regierung, eine nationale Normungsstrategie zu implementieren und chinesische Standards, vor allem bei Zukunftstechnologien, entlang der Seidenstraße sowie weltweit voranzutreiben. Offiziell veröffentlicht ist bislang ein Arbeitsprogramm für die nationale Normungsarbeit, das bis 2025 reicht, sich aber bis 2035 auswirken soll (SAC, 2021). Als Vehikel für die vorrangig betriebene technologische Standardisierung nach chinesischem Muster dienen zum einen die bilateralen Abkommen der Volksrepublik mit ihren Partnern entlang der Digital Silk Road. Zum anderen besetzen chinesische Unternehmen zunehmend die internationalen Standardisierungsinstitutionen, während der Westen darin an Einfluss verliert. Dazu zählen neben der Internationalen Elektrotechnischen Kommission (IEC) und der Internationalen Fernmeldeunion (ITU) auch die Internationale Organisation für Normung (ISO). Sie ist für Produktnormen zuständig und Deutschland war hier bisher sehr gut vertreten (SAC, 2021; Arcesati, 2020; Rühlig, 2020). Das könnte sich schnell ändern.

Nur erwähnt seien schließlich die „Health Silk Road“, die „Space Silk Road“ und die „Educational Silk Road“ (z. B. Mardell, 2020b; Lew et al., 2021). Weitere Initiativen, die Chinas weltweiten Führungsanspruch untermauern sollen, dürften folgen.

4 Dies hätte 2019 bei einem Bruttoweltprodukt von 88 Billionen US-$ (Worldbank, 2021) rund 1,3 Billionen US-$ entsprochen.

**Title:** ***China's Belt and Road Initiative Is Changing***

**JEL Classification:** *F21, O22, O53*
